# Associations of hand-washing frequency with incidence of acute respiratory tract infection and influenza-like illness in adults: a population-based study in Sweden

**DOI:** 10.1186/1471-2334-14-509

**Published:** 2014-09-18

**Authors:** Hanna Merk, Sharon Kühlmann-Berenzon, Annika Linde, Olof Nyrén

**Affiliations:** Department of Medical Epidemiology and Biostatistics, Karolinska Institutet, Stockholm, Sweden; Department of Analysis and Prevention, Swedish Institute for Communicable Disease Control, Solna, Sweden; Swedish Institute for Communicable Disease Control, Solna, Sweden; Department of Medicine, Vanderbilt University School of Medicine, Nashville, Tennessee USA; Enheten för Epidemiologi och Hälsoekonomi, Avdelningen för Epidemiologi och Uppföljning, Folkhälsomyndigheten, SE-171 82 Solna, Sweden

**Keywords:** Hand hygiene, Common cold, Epidemiology, Public health

## Abstract

**Background:**

Frequent hand-washing is standard advice for avoidance of respiratory tract infections, but the evidence for a preventive effect in a general community setting is sparse. We therefore set out to quantify, in a population-based adult general population cohort, the possible protection against acute respiratory tract infections (ARIs) conferred by a person’s self-perceived hand-washing frequency.

**Methods:**

During the pandemic influenza season from September 2009 through May 2010, a cohort of 4365 adult residents of Stockholm County, Sweden, reported respiratory illnesses in real-time. A questionnaire about typical contact and hand-washing behaviour was administered at the end of the period (response rate 70%).

**Results:**

There was no significant decrease in ARI rates among adults with increased daily hand-washing frequency: Compared to 2–4 times/day, 5–9 times was associated with an adjusted ARI rate ratio (RR) of 1.08 (95% confidence interval [CI] 0.87-1.33), 10–19 times with RR = 1.22 (CI 0.97-1.53), and ≥20 times with RR = 1.03 (CI 0.81-1.32). A similar lack of effect was seen for influenza-like illness, and in all investigated subgroups. We found no clear effect modification by contact behaviour. Health care workers exhibited rate ratio point estimates below unity, but no dose-risk trend.

**Conclusions:**

Our results suggest that increases in what adult laymen perceive as being adequate hand-washing may not significantly reduce the risk of ARIs. This might have implications for the design of public health campaigns in the face of threatening outbreaks of respiratory infections. However, the generalizability of our results to non-pandemic circumstances should be further explored.

**Electronic supplementary material:**

The online version of this article (doi:10.1186/1471-2334-14-509) contains supplementary material, which is available to authorized users.

## Background

Acute respiratory tract infections (ARI) constitute a large part of the total disease burden among people in productive ages and cause significant costs for society [1, 2]. Hand-washing is typically recommended as a central non-pharmacological measure against ARI, but the evidence for its effectiveness is surprisingly sparse, weak and divergent [3]. Cluster-randomized non-blinded intervention trials, sometimes combining hand hygiene with wearing of face masks, in households with newly infected index cases generally failed to statistically confirm protective effects on primary outcomes [3–5], but some found significant protection in subgroups that started intervention early [5, 6]. Public health intervention trials with cluster-randomization in more or less confined population segments have yielded a similar overall pattern of results with a mix of positive studies [3, 7–9] and studies with non-significant effects on the primary outcome but some positive findings in subgroup analyses [3, 10–13]. Almost unavoidable limitations, including Hawthorne effects and outcome information bias, make the results unpersuasive. Graphs showing crude data of disease occurrence over time in positive studies are unconvincing [7, 8]. Hospital-based case–control studies of SARS and severe influenza, limited by their inherent uncertainty as to the controls’ representativity of the population strata that generated the cases, and the possibility of recall bias, provide the most consistent support for a protective effect of frequent hand-washing [3, 14]. Prospective cohort studies have yielded mixed results [3].

To shed further light on the debated [15, 16] value of healthy individuals’ regular hand-washing, we applied a previously validated methodology [17] in a population-based adult general population cohort, in order to quantify the association between self-reported frequency of what is perceived as adequate hand-washing and ARI incidence.

## Methods

### Study design and setting

The Swedish Institute for Communicable Disease Control (SMI) runs a population-based, passive influenza surveillance system in Stockholm County, Sweden since 2007 [17, 18]. Selected individuals, drawn at random from continuously updated population registers, are asked to report, on their own accord, all colds and fevers within one week of onset, using a toll-free interactive voice response (IVR) telephone service or a secure web site. The reports consist of automated tree-structured interviews about the presence/absence of 14–16 specified symptoms. The participants in this analysis were recruited in August and September 2009 using random sampling from the source population supplemented by invitations to participants from the previous season. They were followed up through May 2010, i.e. during the season when influenza A(H1N1)pdm09 struck Sweden. Questionnaires about contact behaviours and typical hand-washing frequency were distributed to the participants between April 12 and May 3, 2010, either through e-mails with a personal link to a web questionnaire, or via regular mail with a paper form. One reminder was sent in the following month.

### Participants

Surveillance participants, aged 17–95 years and who returned a questionnaire, were considered for the analysis. In order to avoid reverse causation, as exposure assessment was performed towards the end of the follow-up period, we excluded those who reported onset of a disease episode within 14 days before receipt of the questionnaire or after receiving the questionnaire but before the questionnaire was returned.

### Exposure definitions

The typical frequency of hand-washing was self-estimated through the following question: “During a regular weekday last week how often did you wash your hands?” The response alternatives were categorized into 0, 1, 2–4, 5–9, 10–19 and ≥20 times. If participants considered the activities in the previous week to be markedly different from those in a regular week, they were instructed to let their answers reflect their most recent “ordinary” week. Age (categorized into 10-year bands) and gender were derived from the National Registration Number, while all other covariates (overall close contact frequency – the number of different people who remained at a distance of <1 meter during >1 minute during a regular weekday in the most recent “ordinary” week; close contacts with children under the age of 13; highest attained education; household size; vaccination against pandemic and or seasonal influenza after September 1, 2009; occupational status; work with patients in health care or related areas) were self-reported and categorized as shown in Table 1. Questions were further asked about various specific aspects of personal close contacts (work-related, long duration [>5 min], physical), but these variables were only used to refine model fit and are not detailed here. Smoking did not meet our criteria for confounding in simple models including age and overall close contact. More specifically, smoking showed an association to the outcome that was considered too weak for inclusion in the models (rate ratio (RR) >0.7 but <1.3). Thus, smoking was not included in our models.

**Table 1:** **Individuals by typical hand-washing frequency and sociodemographic characteristics among participants (n = 2963) providing valid exposure information**

Variable	Grouping	Typical hand-washing frequency (times per day)						
		0-1	2-4	5-9	10-19	≥20	Missing info	Total
		Number of individuals (row percentages)						
Gender	Male	16 (1%)	230 (21%)	516 (47%)	186 (17%)	121 (11%)	29 (3%)	1098 (100%)
	Female	5 (0.3%)	121 (6%)	665 (36%)	582 (31%)	443 (24%)	49 (3%)	1865 (100%)
Age (years)	17-26	2 (2%)	23 (19%)	59 (49%)	18 (15%)	17 (14%)	2 (2%)	121 (100%)
	27-46	5 (1%)	79 (11%)	269 (37%)	214 (29%)	135 (19%)	25 (3%)	727 (100%)
	47-66	8 (1%)	125 (10%)	467 (38%)	339 (27%)	269 (22%)	27 (2%)	1235 (100%)
	≥67	6 (1%)	124 (14%)	386 (44%)	197 (22%)	143 (16%)	24 (3%)	880 (100%)
Occupational status^1^	A^1^	8 (1%)	158 (11%)	565 (38%)	400 (27%)	329 (22%)	29 (2%)	1489 (100%)
	B^1^	8 (1%)	152 (13%)	496 (44%)	271 (24%)	172 (15%)	30 (3%)	1129 (100%)
	C^1^	5 (2%)	37 (13%)	108 (37%)	84 (29%)	53 (18%)	5 (2%)	292 (100%)
	Missing info	0 (0%)	4 (8%)	12 (23%)	13 (25%)	10 (19%)	14 (26%)	53 (100%)
Education (years)	< 9	2 (1%)	35 (11%)	131 (41%)	91 (28%)	54 (17%)	10 (3%)	323 (100%)
	9-13	10 (1%)	116 (12%)	379 (40%)	230 (24%)	203 (21%)	21 (2%)	959 (100%)
	>13	9 (1%)	189 (12%)	621 (40%)	418 (27%)	282 (18%)	27 (2%)	1546 (100%)
	Missing info	0 (0%)	11 (8%)	50 (37%)	29 (21%)	25 (19%)	20 (15%)	135 (100%)
Vaccination	No	10 (1%)	106 (12%)	354 (40%)	212 (24%)	183 (21%)	22 (2%)	887 (100%)
	Yes^2^	11 (1%)	242 (12%)	820 (40%)	552 (27%)	380 (19%)	43 (2%)	2048 (100%)
	Missing info	0 (0%)	3 (11%)	7 (25%)	4 (14%)	1 (4%)	13 (46%)	28 (100%)
Household size (persons)	1	9 (1%)	112 (14%)	331 (42%)	178 (23%)	136 (17%)	22 (3%)	788 (100%)
	2	7 (1%)	153 (11%)	528 (39%)	356 (26%)	278 (21%)	26 (2%)	1348 (100%)
	≥3	5 (1%)	86 (11%)	318 (39%)	231 (29%)	146 (18%)	23 (3%)	809 (100%)
	Missing info	0 (0%)	0 (0%)	4 (22%)	3 (17%)	4 (22%)	7 (39%)	18 (100%)
Overall close contacts	0-4	4 (1%)	97 (19%)	230 (46%)	105 (21%)	54 (11%)	8 (2%)	498 (100%)
	5-19	8 (1%)	160 (13%)	516 (43%)	300 (25%)	185 (16%)	21 (2%)	1190 (100%)
	≥20	9 (1%)	86 (8%)	394 (35%)	332 (29%)	300 (27%)	8 (1%)	1129 (100%)
	Missing info	0 (0%)	8 (5%)	41 (28%)	31 (21%)	25 (17%)	41 (28%)	146 (100%)
Close contacts with children <13 years	0	6 (1%)	161 (14%)	519 (44%)	258 (22%)	205 (18%)	22 (2%)	1171 (100%)
	1-4	10 (1%)	136 (12%)	451 (41%)	296 (27%)	187 (17%)	10 (1%)	1090 (100%)
	≥5	4 (1%)	37 (7%)	152 (31%)	164 (33%)	135 (27%)	6 (1%)	498 (100%)
	Missing info	1 (0.5%)	17 (8%)	59 (29%)	50 (25%)	37 (18%)	40 (20%)	204 (100%)
Healthcare work	No	9 (1%)	181 (12%)	623 (41%)	424 (28%)	259 (17%)	28 (2%)	1524 (100%)
	Yes	2 (1%)	7 (3%)	33 (16%)	45 (22%)	116 (57%)	2 (1%)	205 (100%)
	Missing info	10 (1%)	163 (13%)	525 (43%)	299 (24%)	189 (15%)	48 (4%)	1234 (100%)
TOTAL		21 (1%)	351 (12%)	1181 (40%)	768 (26%)	564 (19%)	78 (3%)	2963 (100%)

^1^A = Gainfully employed; B = Retired/long term sick leave; C = Student/Other.

^2^For seasonal or pandemic flu after September 2009.

### Outcome definition

Proceeding from the symptoms reported, disease events were classified as ARI and influenza-like illness (ILI) based on case definitions proposed by the Commission of the European Communities [19].

### Statistical methods

The associations between typical frequency of hand-washing and incidence of ARI and ILI episodes, respectively, were modelled using negative binomial regression with rate ratios as the measure of effect [20]. We postulated *à priori* that age, gender, educational level and vaccination status were plausible confounding factors and thus qualified as covariates in all multivariable models. In a simple model including age and overall close contact, occupational status met the criteria for confounding with the outcome ARI but not ILI and was included accordingly. In our main analysis we considered interaction between included contact-related covariates and hand-washing in a stepwise forward manner. The contact-related covariates were considered as possible effect modifiers since they could affect the number of infections encountered. Model fit was evaluated using likelihood-ratio tests [21] and the candidate interactions that were statistically significant were further estimated in strata.

We carried out subanalyses with restriction to (1) gainfully employed participants; (2) employed, studying or other not working in the health care sector; (3) participants who were unvaccinated against the seasonal and pandemic influenza; (4) the high season for influenza (September 28, 2009 to December 27, 2009, based on laboratory surveillance data); and (5) the post-peak season (December 28, 2009 to May 23, 2010).

A previous validation study of the surveillance system found that it missed 60% of ARI and fever episodes whereas the false reporting of ARI and fever episodes was just 1% [17]. To evaluate possible effects of participants’ underreporting, we conducted a series of sensitivity analyses of the main model correcting for underreporting. For each age group we calculated the total number of unreported ARI reports (*m*) based on the total number of person-weeks and number of reported ARIs in the surveillance system, and on the negative predictive value (NPV). We randomly selected *m* participants with replacement and assigned them an ARI report every time they were selected. The corrected number of ARI reports was the number of reports in the surveillance system plus the number of unreported ARIs. The entire procedure was repeated 20 times for each age group, each time with a different NPV randomly obtained from the 95% confidence interval from the validation study. The covariate adjusted main model was then fitted using the 20 corrected datasets. Three different ways of selecting the participants when assigning the missing reports were applied to create sensitivity analyses based on different assumptions. In one the participants were selected with probability weights proportional to the number of ARIs the participant had reported to the system; those with no reports were assumed to have a weight of 0.5. In another scenario the probability weight was inverse to the number of ARI reports, and those with no reports were assumed to have a weight of 2; and in the last all participants had the same weights.

STATA 12 (StataCorp; USA) was used for all statistical analyses except for the derivation of the datasets for the sensitivity analysis for which we used R 3.0.0. P-values <0.05 were considered significant, except for the likelihood ratio tests for interaction where we chose a more conservative significance level, p < 0.20.

### Ethics

Ethics approval was granted by the Stockholm Regional Research Ethics Review Board (2010/237-31/5). Actively registering in the surveillance system and returning a questionnaire was considered as informed consent.

## Results

**Figure 1:**
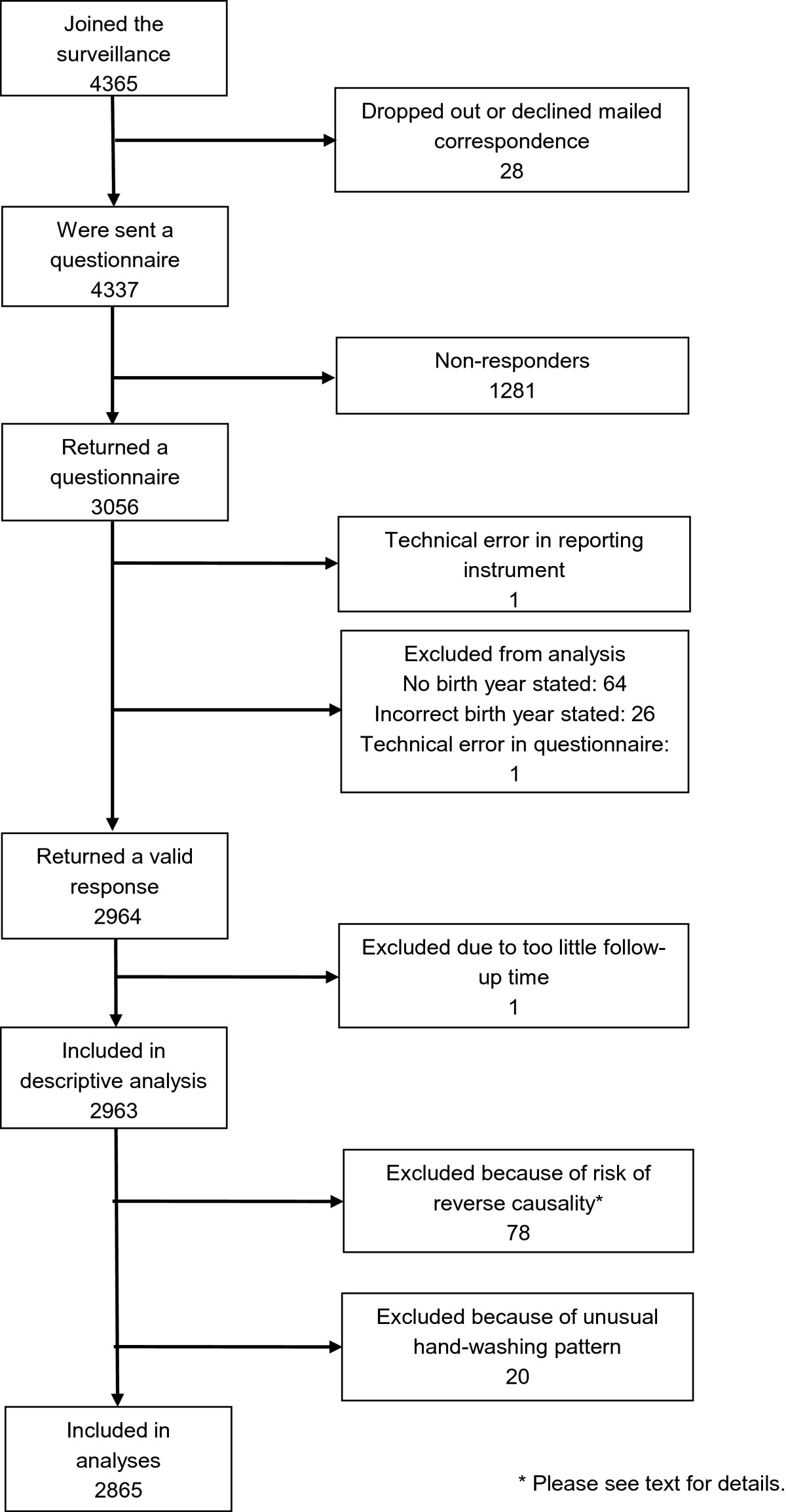
**The passage of adult participants through the study.**

Of 4337 adult participants, 3056 (70.5%) returned completed questionnaires (Figure 1), but 92 were excluded due to technical errors or uncertainty about the identity of the responder. To avoid reverse causality, as discussed in the methods section, another 78 were excluded. Only 20 of the remaining participants reported hand-washing ≤1/day. This category was too small to fit in our multivariable models, and was considered too deviant to include in the reference category (2–4 hand-washings/day). Not washing hands as opposed to washing hands 4 times per day may have very different effects, grouping together the categories hand-washing ≤1/day and 2-4/day may even produce significant but misleading results. Therefore, out of prudence, we chose to exclude from all our analyses participants who reported hand-washing ≤1/day.

Most participants lived in 1-2-person households (Table 1), 50% were gainfully employed and more than half had >13 years of formal education. Only 10% were daily smokers. Owing to a concurrent national immunization campaign against influenza A(H1N1)pdm09 with 60% coverage [22], only 30% of the participants were unvaccinated against the seasonal and/or pandemic influenza. Most participants (85%) washed their hands 5 times/day or more.

Our participants differed from the source population (N = 1,604,036) in regard to gender (63% versus 51% were women) and age (mean 56.4 versus 46.3 years) [23]. Participants who did not respond to the questionnaire were, on average, younger than responders. The gender distribution among non-responders (54% women) was less skewed, and significantly different from responders (p < 0.001). Of participants who answered the questionnaire and those who did not 38% and 21% (p < 0.001), respectively, reported ≥1 ARI. Yet, the associations of age and sex with ARI incidence followed a similar pattern in both categories (in Additional file 1: Table S1).

With mean follow-up time of 36 weeks, the participants contributed 105,526 person-weeks under surveillance. Missing data was ≤7% for all variables. Increasing hand-washing frequency above 2–4 times daily was not associated with a reduction in the incidence of respiratory tract infection in the crude analysis. On the contrary, overall it was associated with a moderately increased incidence of both ARI and ILI up through the second highest exposure category (10–19 times per day), whereupon the relative risk fell somewhat (Table 2). The multivariably adjusted excess risk was only borderline significant for ARI among those washing hands 10–19 times per day, and non-significant for ILI. With ARI as the outcome, there was no interaction between hand-washing frequency and, respectively, household size (p = 0.24), overall contact frequency (p = 0.52) or child contact frequency (p = 0.49). For ILI, hand-washing tended to interact with household size (p = 0.09). Among subjects living in households with 3 members or more, there was a slight, statistically non-significant tendency for point estimates of relative risk of ILI to be below unity in categories with frequent hand-washing, however without any clear dose-risk pattern (Table 3).

**Table 2:** **Self-reported acute respiratory tract infection and influenza-like illness rate ratios by hand-washing habits with 95% confidence intervals**

	Acute respiratory tract infection		Influenza-like illness	
Hand-washing frequency	Crude	Adjusted^1^	Crude	Adjusted^2^
2-4 times daily	1 (reference)	1 (reference)	1 (reference)	1 (reference)
5-9 times daily	1.14 (0.93-1.40)	1.08 (0.87-1.33)	1.10 (0.75-1.59)	0.98 (0.66-1.46)
10-19 times daily	1.37 (1.11-1.69)	1.22 (0.97-1.53)	1.48 (1.01-2.16)	1.25 (0.82-1.90)
≥20 times daily	1.16 (0.93-1.45)	1.03 (0.81-1.32)	1.23 (0.82-1.85)	1.06 (0.68-1.67)

^1^adjusted for age, vaccination status, gender, educational level, occupational status, household size, overall and child contact.

^2^adjusted for age, gender, educational level, vaccination status, household size, overall and child contact.

**Table 3:** **Relative risks (rate ratios with 95% confidence intervals) for self-reported influenza-like illness (ILI) stratified by household size and acute respiratory tract infection (ARI) stratified by health care work, both by different hand-washing frequency**

Subset and strata		Typical hand-washing frequency				
	n	2-4 times daily	5-9 times daily	10-19 times daily	≥20 times daily	Interaction p-value
**Among all responding participants, outcome = ILI**		Rate ratio (95% confidence interval)				
Household size 1 person^1^	639	1 (reference)	0.69 (0.33-1.45)	1.89 (0.91-3.91)	1.04 (0.45-2.38)	0.09
Household size 2 persons^1^	1137	1 (reference)	1.16 (0.62-2.19)	1.27 (0.66-2.46)	1.25 (0.63-2.50)	
Household size ≥3 persons^1^	706	1 (reference)	1.03 (0.52-2.04)	0.88 (0.43-1.82)	0.89 (0.41-1.93)	
**Among gainfully employed participants, outcome = ARI**						
Non-health care work^2^	1104	1 (reference)	1.20 (0.85-1.69)	1.39 (0.97-2.00)	1.19 (0.80-1.76)	0.13
Health care work^2^	170	1 (reference)	0.31 (0.11-0.90)	0.49 (0.18-1.31)	0.45 (0.18-1.13)	

^1^adjusted for age, gender, educational level, vaccination status, child- and overall contact, household size.

^2^adjusted for age, gender, educational level, vaccination status, household size, physical-, child-, and long contacts, and health care work status.

Subanalyses restricted to people unvaccinated against influenza, non-health care workers, to the influenza high season, to the influenza post-peak season, and to gainfully employed individuals all showed essentially the same pattern of slightly increasing risk with increasing hand-washing frequency up to the second highest category, but none of the point estimates attained statistical significance (in Additional file 2: Table S2). In the latter subanalysis there was some indication of interaction between hand-washing and health care work, but only for ARI (p = 0.13) and not for ILI (p = 0.52). Among 170 participants with health care work, the risk of ARI was 69% (95% CI 10-89%) lower among those who washed their hands 5–9 times per day, compared to those with only 2–4 hand-washes per day, but the risk reduction was less impressive (point estimates 51-55%), statistically non-significant and without a clear dose-risk pattern in categories with hand-washing frequency exceeding 9 times per day (Table 3).

None of our sensitivity analyses correcting for underreporting of the outcome changed the result of the main analysis, namely that an increasing frequency of hand-washing was not associated with any decreases in ARI incidence (in Additional file 3: Table S3).

## Discussion

The overall results of this population-based prospective observational study with adjustments for contact behaviour provide no support for the notion that own habitual hand-washing above 4 times daily confers protection to the hand-washing individual against respiratory tract infection. It should be emphasized that hand-washing was self-defined and the cleansing probably ranged from quick rinses to the recommended 40–60 seconds washing with soap and drying of hands [24]. A subanalysis suggested a protective effect among health care workers, who are more likely to follow existing recommendations. Therefore, the absence of an overall protective effect of hand-washing in our population-based sample might perhaps be explained in part by inadequate thoroughness of the cleaning, but since this study only collected information about frequency and not about thoroughness, it does not provide the data needed to support or reject this speculation.

Our negative results may seem counterintuitive and in conflict with the existing literature. However, although many studies conclude that hand-washing should be recommended as a public health measure in the face of threatening upper respiratory tract virus epidemics, the scientific evidence remains unconvincing. To a large extent, the evidence is derived from studies among children [3], in whom the baseline hand hygiene is often imperfect and the interventions are typically enforced by dedicated adults, in non-blinded cluster-randomized trials [3–13] with risks for Hawthorne effects and biased outcome assessment, or in hospital-based case–control studies [3, 14] with their known risk of bias. Moreover, despite the meticulous design of most intervention studies, many of them confirmed a statistically significant effect on the main outcome only in subgroup analyses [6, 10–13] or the effect was confined to parts of the observation period [7, 8].

A recent Finnish cluster-randomized intervention study found a significant effect of hand-hygiene with soap and water on ARI occurrence, but when the influenza A(H1N1) 2009 pandemic struck, a concurrent nationwide campaign for improved hand hygiene seemingly annulled differences between the intervention arms [8]. Since our study coincided with the influenza pandemic in Sweden we cannot exclude the possibility that temporary changes in hand hygiene habits may have attenuated possible protective effects of the reported habitual washing pattern. However, subanalyses in the influenza high-season and the post-peak season did not reveal any important differences.

Interaction between hand-washing and contact behaviour was occasionally suggested in our models, but estimation of effects in strata did not support any important effect modification by contact pattern. The lack of effect of hand-washing was similar for both ARI and ILI, and subanalyses among participants who were unvaccinated against influenza yielded results comparable to those in the total cohort, thus refuting the assumption that widespread vaccination in the population had made hand hygiene less important.

While insufficient thoroughness of what lay people perceive as adequate hand-washing is an attractive explanation for our negative results, other alternative hypotheses may need to be formulated to reconcile our own results with those in the combined literature. One is that most ARIs among adults spread mainly via the airborne route and less via direct or indirect contact. Another is that frequent hand-washing is more efficient on the population level when it is practiced by those who are already sick, and less efficient when practiced by healthy individuals. The observed tendency towards increased risk among individuals who habitually wash their hands 10–19 times/day might even be the result of false security, or even more speculatively, a perturbed protective skin microbiota [25, 26].

Strengths of our study include the prospective population-based design, the large sample size, and the adjustments for socioeconomic status and contact behaviours.

Notable limitations include the low participation in the surveillance cohort; although the response rate for the contact and hand-washing questionnaire was high among those who participated in the surveillance, only 44% of the invited individuals (including guardians of children) joined the surveillance cohort. It is reasonable to assume that members of the surveillance cohort were, on average, more health conscious than the average individual in the population. The high vaccination coverage might signal such health consciousness. If they zealously engaged in other preventive measures, e.g. avoiding contacts with sick people, the scope for an effect of hand-washing might have been limited. On the other hand, this selected group probably also conscientiously reports ARI symptoms and thus strengthens the internal validity of the study. Additionally, since the group with no, or next to no, hand-washing was too small to serve as a separate category, let alone as reference in our analyses, we could not shed light on whether hand-washing had an effect on ARI risk compared to no hand-washing. The possibility of threshold effects in the lowest range of hand-washing activity must be entertained as must the possibility of effects on specific infectious agents as opposed to the syndromes measured in this study. Another important limitation is in the predictably substantial underreporting of the infectious outcomes [17]. Underreporting that is non-differential vis-à-vis the explanatory variables will not bias the risk-ratio estimate if the proportion with false positive reports is negligible [27]. A previous validation study confirmed that the false positive reporting is indeed insignificant [17], but we cannot exclude the possibility that the underreporting was differential, i.e. that participants who washed their hands frequently were more prone to report disease to the surveillance system. The study could also be affected by social desirability bias, i.e. those who infrequently washed their hands exaggerated their hand-washing frequency more than those who frequently washed their hands, and this could have obscured the effect of hand-washing. Furthermore, despite a high response rate of 70.5%, it is possible that the non-responders could have experienced an effect of hand-washing on their ARI risk which we were not able to study.

## Conclusions

We conclude, although with a number of caveats, that an increasing frequency of self-defined hand-washes among healthy individuals does not seem to be associated with a decreasing incidence of ARIs or ILIs. Since health-care workers seemingly constituted a possible exception, the lack of effect in non-health care workers might be explained by insufficiently thorough hand-washes. This interpretation can only be substantiated in specially designed studies, but it may have important implications for the design of future public health interventions. The generalizability of the study results needs to be explored further, during non-pandemic circumstances and in other populations.

## Electronic supplementary material

Additional file 1: Table S1: Crude negative binomial regression modelling of relative risks of self-reported acute respiratory tract infection in age and gender groups by responders, who provided adequate follow-up time, (n=2,963) and non-responders (n=1,373) to the questionnaire about contact behaviours and typical hand-washing frequency. Rate ratios with 95% confidence intervals. (PDF 50 KB)

Additional file 2: Table S2: Crude and multivariable negative binomial regression modelling of the relative risks by hand-washing habits in subgroups. Self-reported acute respiratory tract infection and influenza-like illness rate ratios with 95% confidence intervals including the statistically indicated interaction by health care work among gainfully employed. (PDF 61 KB)

Additional file 3: Table S3: Sensitivity analysis of the estimates of relative risk of self-reported acute respiratory tract infection (ARI) by hand-washing habits using corrected datasets. (PDF 72 KB)

Below are the links to the authors’ original submitted files for images.

Authors’ original file for figure 1

## Abbreviations

ARI: Acute respiratory tract infection
CI: Confidence interval
ILI: Influenza-like illness
IVR: Interactive voice response
*m*: The total number of unreported ARI reports
NPV: Negative predictive value
RR: Rate ratio
SMI: Swedish Institute for Communicable Disease Control.
