# Supplementary material for: Associations of hand-washing frequency with incidence of acute respiratory tract infection and influenza-like illness in adults: a population-based study in Sweden
Source: BMC Infect Dis. 2014 Sep 18;14:509. doi: 10.1186/1471-2334-14-509 (PMC4177698; doi:10.1186/1471-2334-14-509)
Supplement: Supplementary file 1 — Additional file 1: Table S1: Crude negative binomial regression modelling of relative risks of self-reported acute respiratory tract infection in age and gender groups by responders, who provided adequate follow-up time, (n=2,963) and non-responders (n=1,373) to the questionnaire about contact behaviours and typical hand-washing frequency. Rate ratios with 95% confidence intervals. (PDF 50 KB) [file 12879_2014_3827_MOESM1_ESM.pdf]

Associations of hand-washing Frequency with Incidence of Acute Respiratory Tract Infection and Influenza-like Illness in Adults: a population-based Study in Sweden.
Hanna Merk, Sharon Kühlmann-Berenzon, Annika Linde, Olof Nyrén

Supplementary table 1. Crude negative binomial regression modelling of relative risks of self-reported acute respiratory tract infection in age and gender groups by responders, who provided adequate follow-up time, (n=2,963) and non-responders (n=1,373) to the questionnaire about contact behaviours and typical hand-washing frequency. Rate ratios with 95% confidence intervals.

		Respondents	Non-respondents
Age (years)	17-26	1 (reference)	1 (reference)
	27-36	0.92 (0.69-1.22)	0.87 (0.57-1.31)
	37-46	0.76 (0.58-1.01)	0.75 (0.49-1.13)
	47-56	0.63 (0.48-0.83)	0.77 (0.49-1.22)
	57-66	0.69 (0.53-0.89)	0.86 (0.56-1.31)
	67-76	0.61 (0.47-0.81)	0.66 (0.40-1.08)
	77-86	0.56 (0.41-0.77)	0.65 (0.35-1.21)
	87-96	0.27 (0.12-0.57)	0.20 (0.03-1.59)
Gender	Male	1 (reference)	1 (reference)
	Female	1.40 (1.25-1.58)	1.23 (0.96-1.56)
