# Supplementary material for: Associations of hand-washing frequency with incidence of acute respiratory tract infection and influenza-like illness in adults: a population-based study in Sweden
Source: BMC Infect Dis. 2014 Sep 18;14:509. doi: 10.1186/1471-2334-14-509 (PMC4177698; doi:10.1186/1471-2334-14-509)
Supplement: Supplementary file 2 — Additional file 2: Table S2: Crude and multivariable negative binomial regression modelling of the relative risks by hand-washing habits in subgroups. Self-reported acute respiratory tract infection and influenza-like illness rate ratios with 95% confidence intervals including the statistically indicated interaction by health care work among gainfully employed. (PDF 61 KB) [file 12879_2014_3827_MOESM2_ESM.pdf]

Associations of hand-washing Frequency with Incidence of Acute Respiratory Tract Infection and Influenza-like Illness in Adults: a population-based Study in Sweden.

Hanna Merk, Sharon Kühlmann-Berenzon, Annika Linde, Olof Nyrén

Supplementary table 2. Crude and multivariable negative binomial regression modelling of the relative risks by hand-washing habits in subgroups. Self-reported acute respiratory tract infection and influenza-like illness rate ratios with 95% confidence intervals including the statistically indicated interaction by health care work among gainfully employed.

			Hand washing RR (95% CI)					
	Outcome	n	0-1 times daily	2-4 times daily	5-9 times daily	10-19 times daily	≥20 times daily	Interaction p-value
**Gainfully employed participants [I]**								
Crude	ARI	1396	Omitted	1	1.19 (0.87-1.63)	1.42 (1.03-1.95)	1.28 (0.92-1.79)	
Adjusted[1]	ARI	1274	Omitted	1	1.07 (0.77-1.47)	1.26 (0.90-1.77)	1.09 (0.76-1.57)	
Adjusted non-hcw[1]	ARI	1104	Omitted	1	1.20 (0.85-1.69)	1.39 (0.97-2.00)	1.19 (0.80-1.76)	0.13
Adjusted hcw[1]	ARI	170	Omitted	1	0.31 (0.11-0.90)	0.49 (0.18-1.31)	0.45 (0.18-1.13)	
Crude	ILI	1396	Omitted	1	1.30 (0.74-2.31)	1.50 (0.84-2.70)	1.54 (0.85-2.80)	
Adjusted[1]	ILI	1274	Omitted	1	1.11 (0.62-2.01)	1.38 (0.74-2.55)	1.41 (0.73-2.70)	
**Non-hcw (studying/other and employed)[II]**								
Crude	ARI	1446	Omitted	1	1.19 (0.89-1.59)	1.38 (1.02-1.87)	1.21 (0.87-1.68)	
Adjusted[2]	ARI	1317	Omitted	1	1.12 (0.83-1.50)	1.26 (0.91-1.73)	1.10 (0.78-1.56)	
Crude	ILI	1446	Omitted	1	0.97 (0.60-1.57)	1.07 (0.65-1.78)	0.92 (0.53-1.61)	
Adjusted[3]	ILI	1329	Omitted	1	0.84 (0.51-1.39)	0.94 (0.55-1.60)	0.87 (0.48-1.57)	
**Unvaccinated[III *]**								
Crude	ARI	828	Omitted	1	0.98 (0.69-1.39)	1.16 (0.80-1.69)	1.16 (0.79-1.69)	
Adjusted[4]	ARI	722	Omitted	1	1.02 (0.71-1.47)	1.18 (0.79-1.75)	1.12 (0.74-1.70)	
Crude	ILI	828	Omitted	1	0.99 (0.53-1.83)	1.43 (0.75-2.70)	0.97 (0.49-1.92)	
Adjusted[5]	ILI	731	Omitted	1	1.01 (0.53-1.94)	1.38 (0.70-2.72)	0.97 (0.46-2.05)	
**During influenza high season[#]**								
Crude	ARI	2788	Omitted	1	1.11 (0.84-1.46)	1.31 (0.99-1.75)	1.00 (0.73-1.36)	
Adjusted[4]	ARI	2462	Omitted	1	1.04 (0.78-1.39)	1.12 (0.82-1.52)	0.87 (0.62-1.22)	
Crude	ILI	2788	Omitted	1	1.05 (0.64-1.72)	1.39 (0.84-2.30)	1.00 (0.57-1.74)	
Adjusted[5]	ILI	2482	Omitted	1	0.98 (0.59-1.63)	1.20 (0.70-2.07)	0.98 (0.54-1.77)	
**During influenza post-peak season[IV ##]**								
Crude	ARI	2787	Omitted	1	1.03 (0.76-1.38)	1.31 (0.97-1.78)	1.13 (0.81-1.56)	
Adjusted[4]	ARI	2461	Omitted	1	0.93 (0.68-1.27)	1.14 (0.81-1.59)	0.97 (0.68-1.39)	
Crude	ILI	2787	Omitted	1	1.11 (0.60-2.04)	1.53 (0.82-2.85)	1.37 (0.71-2.65)	
Adjusted[5]	ILI	2481	Omitted	1	0.87 (0.46-1.63)	1.15 (0.59-2.24)	0.99 (0.48-2.04)	

[1] adjusted for age, gender, educational level, vaccination status, household size, physical, child and long contacts, and health care work status

[2] adjusted for age, gender, educational level, vaccination status, household size, child and long contacts, and occupation

Associations of hand-washing Frequency with Incidence of Acute Respiratory Tract Infection and Influenza-like Illness in Adults: a population-based Study in Sweden.

Hanna Merk, Sharon Kühlmann-Berenzon, Annika Linde, Olof Nyrén

[3] adjusted for age, gender, educational level, vaccination status, household size, child and long contacts

[4] adjusted for age, gender, educational level, vaccination status, child and overall contact, household size and occupation

[5] adjusted for age, gender, educational level, vaccination status, child and overall contact, household size

[I] those ≥67 years are excluded, and long contacts are categorized as; 0-4, 5-9 ≥10.

[II] age group 67-76 years and 77-86 years have been combined, and long contacts are categorized as; 0-4, 5-9 ≥10.

[III] age group 67-76, 77-86 and 87-96 years have been combined.

[IV] age group 77-86 and 87-96 years have been combined.

[*] Against seasonal and pandemic flu since September 2009

[#] 28 September - 27 December, 2009

[##] 28 December, 2009 - 23 May, 2010

CI=Confidence interval RR=Rate ratio ARI=Acute respiratory tract infection ILI=Influenza-like illness hcw=health care work
