# Supplementary material for: Associations of hand-washing frequency with incidence of acute respiratory tract infection and influenza-like illness in adults: a population-based study in Sweden
Source: BMC Infect Dis. 2014 Sep 18;14:509. doi: 10.1186/1471-2334-14-509 (PMC4177698; doi:10.1186/1471-2334-14-509)
Supplement: Supplementary file 3 — Additional file 3: Table S3: Sensitivity analysis of the estimates of relative risk of self-reported acute respiratory tract infection (ARI) by hand-washing habits using corrected datasets. (PDF 72 KB) [file 12879_2014_3827_MOESM3_ESM.pdf]

Hanna Merk, Sharon Kühlmann-Berenzon, Annika Linde, Olof Nyrén

Method: The corrected datasets were created by randomly assigning participants' ARI events that had not been reported and adding these events to those reported. For each corrected dataset the number of unreported ARIs was calculated based on results from Merk et al (2013) by age strata and obtained as:

$$m_i = N_i - nARI_i * NPV_i - ARI_i$$

where m_i was the number of unreported ARIs, N_i was the total number of person-weeks; $nARI_i$ was the number non-ARIs (calculated as number of person-weeks with no reported ARI); NPV_i was the negative predictive value; ARI_i was the number of reported ARIs; and i the age stratum. Here we have assumed that there were no false positive ARI reports. In each corrected dataset the NPV_i was selected randomly from its 95% confidence interval, and a new random seed was chosen when assigning the unreported ARI to specific individuals. Different weighting schemes were used to give individuals probabilities of having an unreported ARI assigned to them: equal, proportional and inverse. In the equal weight scheme, all individuals had the same probability i.e. weight; when proportional, the weight was proportional to how many ARIs they had actually reported, and those with no reports were assumed to have a weight of 0.5; and in the inverse proportional, the weights were equal to the inverse of the number of ARIs reported by the individual, those with no reports were assumed to have a weight of 2. For each weight scheme, 20 corrected datasets were created.

For the sensitivity analysis with the equal and proportional weight, we used negative binomial regression to fit the same model as in the original analysis to each iteration; for the inversely weighted data, Poisson regression was used (no overdispersion was seen here). We calculated the mean risk ratio (RR), and the within and in between variance of the RR for each hand washing category over all iterations from the weight scheme. We then calculated the total variance (Little RJA, Rubin DB: *Statistical Analysis with missing data*. Hoboken: John Wiley & Sons; 2002.) and Wald confidence interval of the mean RR for each hand washing category.

In none of the categories could we see a preventive effect mediated by hand-washing. Furthermore, all the differently weighted categories returned results close to unity. This suggests that there was no association between ARI reporting and hand-washing. Had there been an association between underreporting of ARIs and hand-washing, the equally weighted results would have been similar to the main analysis, the proportionally weighted would have shown increased risk ratios and the inverse would have closed in on unity. The inversely weighted data behaved as expected, while the equally and proportionally weighted data did not.

Supplementary table 3. Sensitivity analysis of the estimates of relative risk of self-reported acute respiratory tract infection (ARI) by hand-washing habits using corrected datasets.

	Acute respiratory tract infection RR (95% Confidence intervals)
**Equal weight**	
Hand-washing frequency	Adjusted[1]
2-4 times daily	1 (reference)
5-9 times daily	1.01 (0.88-1.15)
10-19 times daily	1.05 (0.90-1.20)

≥20 times daily	1.01 (0.86-1.16)
**Proportional weight**	
Hand-washing frequency	Adjusted[1]
2-4 times daily	1 (reference)
5-9 times daily	1.04 (0.87-1.22)
10-19 times daily	1.13 (0.93-1.34)
≥20 times daily	1.00 (0.80-1.19)
**Inverse weight**	
Hand-washing frequency	Adjusted[1]
2-4 times daily	1 (reference)
5-9 times daily	1.01 (0.89-1.12)
10-19 times daily	1.02 (0.88-1.15)
≥20 times daily	1.00 (0.86-1.14)

[1] adjusted for age, vaccination status, gender, educational level, occupational status, household size, overall and child contact

RR=Risk ratio
