# Supplementary figures and images for: Associations of hand-washing frequency with incidence of acute respiratory tract infection and influenza-like illness in adults: a population-based study in Sweden
Source: BMC Infect Dis. 2014 Sep 18;14:509. doi: 10.1186/1471-2334-14-509 (PMC4177698; doi:10.1186/1471-2334-14-509)

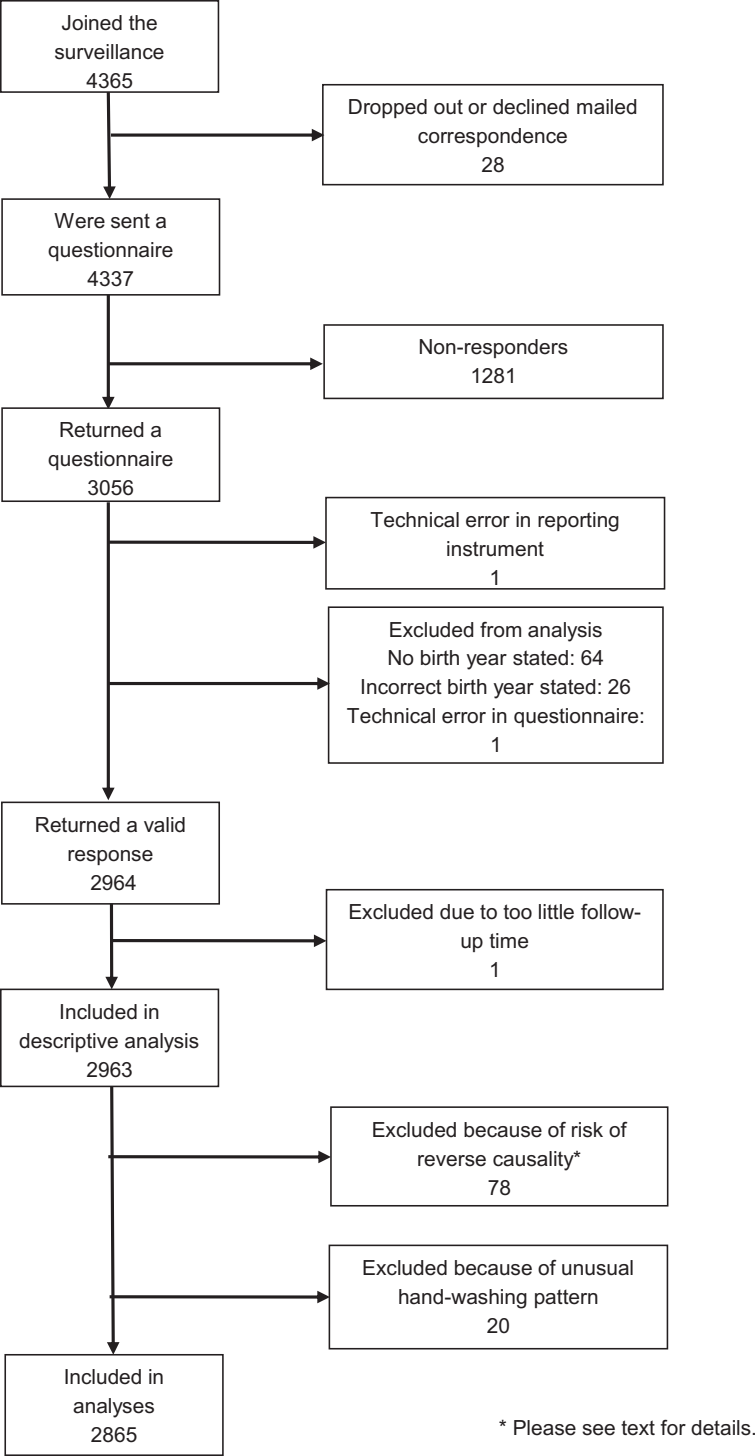

\* Please see text for details.

Supplement: Supplementary file 4 — Authors’ original file for figure 1 [file 12879_2014_3827_MOESM4_ESM.pdf]
